# Examining factors influencing public knowledge and practice of proper face mask usage during the COVID-19 pandemic: a cross-sectional study

**DOI:** 10.7717/peerj.16889

**Published:** 2024-02-08

**Authors:** Vigneshwaran Easwaran, Sultan Alshahrani, Mohammad Jaffar Sadiq Mantargi, Bhavana Bommireddy, Noohu Abdulla Khan, Sirajudeen Shaik Alavudeen, Narayana Goruntla, Tahani Almeleebia, Usman Thattarauthodiyil, Muhammad Awais

**Affiliations:** 1 Department of Clinical Pharmacy, College of Pharmacy, King Khalid University, Abha, Kingdom of Saudi Arabia; 2 Department of Pharmaceutical Sciences, Pharmacy Program, Batterjee Medical College, Jeddah, Saudi Arabia; 3 Department of PharmD, Raghavendra Institute of Pharmaceutical Education and Research, Anantapur, Andhra Pradesh, India; 4 Department of Clinical Pharmacy, School of Pharmacy, Kampala International University, Kampala, Uganda; 5 Department of Musculoskeletal and Sports Injury Rehabilitation, Physiotherapy Program, Batterjee Medical College, Jeddah, Kingdom of Saudi Arabia; 6 Department of Microbiology, General Medicine Practice Program, Batterjee Medical College, Jeddah, Saudi Arabia

**Keywords:** Face masks use, Disposal, Appropriate handling, Public, COVID-19

## Abstract

**Background:**

The COVID-19 pandemic had an enormous impact on people’s quality of life worldwide. Appropriate use of facemasks is an important checkpoint in containing the spread of infection, which was believed to provide the desired level of protection and preserve the community. Given the relative novelty of facemask use in the general population, it is imperative to prioritize the promotion of appropriate facemask utilization and identify factors that may contribute to poor adherence.

**Aim:**

This study assessed the factors that determined facemask use among the public.

**Methods:**

A cross-sectional questionnaire-based study was conducted among the residents of the Kingdom of Saudi Arabia between November 2020 and January 2021. The current study explored the factors such as demographic characteristics influencing the knowledge and practice of proper use of facemasks. The study included a total of 198 participants. The results were derived through binomial logistic regression analysis to determine the relationship between the demographic characteristics and responses.

**Results:**

The key findings of the study which are crucial in developing targeted intervention strategies to enhance the responsible use and disposal of facemasks are gender, income and employment. A significant difference was found between male and female participants regarding a positive approach to using facemasks, such as washing their hands (*P* = 0.042). In addition, homemakers differed significantly from students, regarding the correct usage of facemasks (P = 0.026). The study participants were aware that hand hygiene is essential when putting on and removing facemasks. Despite wearing facemasks properly, adult participants possessed less knowledge about the hazards of reusing facemasks and appropriate disposal (OR = 0.202, 95% CI [0.032–1.298]).

**Conclusion:**

The present research identified gender, income, and employment as the primary attributes that play a pivotal role in the formulation of focused intervention tactics aimed at improving the cautious use and appropriate disposal of facemasks. It is essential to implement nationwide awareness activities, such as information campaigns, to enhance knowledge. Health authorities should establish a functional infrastructure for the collection and disposal of used facemasks by the general public, starting with the dissemination of knowledge. Moreover, the results of the present study have significant implications for health preventive programs aimed at preparing for future pandemics, since they highlight the specific demographic groups that should be prioritized in the development of such policies. Furthermore, it is advisable to integrate these interventional initiatives with national health polices to promote preparedness for handling future pandemics.

## Introduction

The COVID-19 pandemic in the Kingdom of Saudi Arabia accounted for 841,469 confirmed cases of COVID-19 and 9,646 deaths between 3 January 2020 and 6 September 2023 (World Health Organization, 2023). Global data of almost 180 nations as of July 29, 2020 has revealed the following: 16,558,289 were the positive cases reported; with 656,093 deaths; and 25,127 new cases; with a 4.9% mortality rate; and 180 nations afflicted with COVID-19 (Sawicka et al., 2022). However, despite the fact that the COVID-19 pandemic is no longer a public health emergency of global significance, we still need to maintain vaccination campaigns and epidemiological surveillance because of the infection’s current state. The World Health Organization (WHO) declared the emergency on January 30, 2020, and it ended on May 5, 2023, after 3 years and 3 months (Moraga-Llop & Campins-Martí, 2023). The pandemic caused turmoil and substantially impacted the public, including employment uncertainties, financial instability, mental health issues, educational disruption, family well-being concerns, and loss of loved ones. People protected themselves from the spread of the coronavirus by physical and social distancing, lockdowns, and other health protocols such as facemasks and hand hygiene (Gayatri & Puspitasari, 2022). The primary mode of transmission of COVID-19 was clearly through respiratory droplets from the infected individuals, with both symptomatic or asymptomatic individuals prone to transmitting the infection (Lai et al., 2020; Johansson et al., 2021).

Facemasks have long been used traditionally for general infection control. Generally, any pandemic preparedness involves the implementation of both pharmaceutical (vaccination and antiviral drugs) and nonpharmaceutical countermeasures, such as social and physical distancing, hand hygiene, and the use of facemasks (Brienen et al., 2010). Therefore, interventions such as facemasks uses, hand washing, lockdowns, physical distancing, and other hygiene measures were considered major preventive approaches (MacIntyre et al., 2009; Lepelletier et al., 2020). In this regard, facemasks and respirators were strongly recommended by the World Health Organization (WHO) and the Centers for Disease Control and Prevention (CDC) as a standard for transmission-based precaution (Siegel et al., 2007).

The specifications and recommendations regarding how to use facemasks vary across countries, and the use of facemasks has increased after the COVID-19 pandemic, including the use of N95 respirators (without any other protective equipment) (Rahman et al., 2022). Nations worldwide have repeatedly emphasized the necessary measures for the correct utilization of facemasks by the general public. These measures encompass cleansing one’s hands with soap and water or an alcohol-based sanitizer prior to donning the facemask. Additionally, it is important to properly position the facemask to cover the nose, mouth, and chin, ensuring a snug and secure fit. One should refrain from touching the front surface of the facemask while it is being worn, and proper disposal should be conducted (Desai & Mehrotra, 2020; Lee et al., 2020).

The proper facemask removal technique is to hold only the straps, fold the mask inward (possibly several folds) and dispose of it in closed bins with subsequent appropriate hand hygiene (Desai & Mehrotra, 2020; Lee et al., 2020). However, the incorrect use of facemasks might have profound consequences, such as reduced user protection and infection spread to others. In addition, the use of facemasks was unfamiliar, and the public was not accustomed to this practice. Many substandard practices, such as the prolonged use and reuse of facemasks are expected, and there is a paucity of data on this topic (Chughtai, Seale & MacIntyre, 2013). However, it is well known that the appropriate use of facemasks and respirators requires knowledge, training, and supervision (Tang & Wong, 2004; Chughtai et al., 2015). Moreover, individuals’ impudence regarding not being at risk and their lack of awareness concerning the proper use of facemasks might lead to decreased protection against the spread of infections.

The COVID-19 pandemic highlighted the gaps in health surveillance systems, disease prevention, and treatment worldwide (Global Burden of Disease 2021 Health Financing Collaborator Network, 2023). The COVID-19 pandemic has caused significant consequences due to a lack of pandemic preparedness, despite the tremendous research conducted on infectious diseases. This lack of preparedness has resulted in economic damage a substantial loss of human life (Neumann & Kawaoka, 2023).

Past experience demonstrated that many infections reemerge as new outbreaks with and produced high levels of mortality and morbidity. In addition, the identification of new pandemics are being discovered and on the race (Neumann & Kawaoka, 2023)*.*

Facemasks are considered one of the basic preventive measures for any infection transmitted through the respiratory tract. The COVID-19 pandemic has led to increased demand for face masks, particularly the highly infectious omicron variant. Despite vaccinations, most nations still require masks, and the global market for face masks is projected to grow by 4% by 2026. However, improper disposal of masks poses a risk of viral transmission and environmental waste, as they have a limited usage time and must be replaced every 4–10 h (Oludoye et al., 2023). Although facemasks are no longer required during the post**-**pandemic period, their use has become a new normal among the public (Kaewchutima et al., 2023). The prevention of future pandemics may require preparedness from health care agencies and the public. One feasible option for preventing future pandemics is facemasks, and it is essential to educate the general public on the appropriate use and disposal of facemasks (Missoni, Armocida & Formenti, 2021).

A large number of face masks have been disposed of in the environment in recent years, and these masks could release contaminants and may produce ecotoxicological effect (Oliveira et al., 2023). It is a significant public health concern that the improper use of facemasks may lead to not only spread the diseases but also toxicity to the environment (Shammas et al., 2022). Therefore, appropriate disinfection methods should be followed by the public before they dispose of used facemasks. Local health authorities should develop active mechanisms to collect and dispose of used face masks from the public (Asim, Badiei & Sopian, 2021). Additionally, the effective management of used facemasks is necessary to prevent the reemergence of infectious diseases and the emergence of new pathogens. Therefore, the proper management of used facemasks must be encouraged among the public, and is significantly influenced by knowledge and awareness of the public towards the proper usage and disposal of facemasks (Barloa, Lapie & Cruz, 2016; Seng, Fujiwara & Spoann, 2018; Akkajit, Romin & Assawadithalerd, 2020).

Hence, it is essential to explore the existing knowledge, awareness, and practice regarding the appropriate use and disposal of facemasks and to design effective educational intervention (Pappas, 1994; Liang et al., 1999).

There is a scarcity of literature on the factors associated with the suboptimal use of facemasks, and it is imperative to investigate individuals’ willingness and consistency to comply with the guidelines for facemask use. Hence, the current study was conducted with an objective to explore the factors determining facemask use among individuals during the COVID-19 pandemic. This will equip us with valuable insights that will help us successfully navigate future pandemics considering the knowledge, attitude and practice of common people towards facemask usage and its proper disposal, which stands out to be the primary research question of the study.

## Materials & Methods

### Study design, settings, and sample

This was a cross-sectional, questionnaire-based study conducted between November 2020 and January 2021 in Abha City, the capital of the Aseer Region, southwestern Saudi Arabia. The city is situated 2,270 m (7,450 ft) above sea level. The city has the 6th largest population in Saudi Arabia, with a diverse, multiracial, and ethnic population of approximately 376,000. The current study employed the non-probability convenience sampling technique. Irrespective of demographic characteristics, both genders were included if they were 18 years and above to evaluate ecological preservation by studying the proper use and after-use of facemasks at the community level.

### Study procedure

The questionnaires were administered through a web-based platform, and participation was entirely voluntary. All the study participants were duly informed about the study’s purpose and background at the beginning of the questionnaire. No financial or other incentives were provided in exchange for participation. Measures were taken to restrict individuals from taking the survey multiple times, using the provided option in Google Forms to ensure integrity.

A total of 198 completed questionnaires were collected, remaining those which were deemed to be incomplete or irrelevant were excluded in the analysis to keep accuracy, clarity, and consistency. If any respondent not responded to one by third of the items included in the questionnaire were considered incomplete and excluded for the final analysis.

#### Questionnaire and data collection

The questionnaire was prepared referring to published literature and World Health Organization guidelines (Ho, 2012; World Health Organization, 2020; Lee et al., 2020) and reviewed by an expert group. The finalized questionnaire focused on knowledge and attitudes regarding the use and disposal of facemasks. The original questionnaire was prepared in English and translated into Arabic by a team of native Arabic speakers. The language validation was conducted using the retranslation method. The items included in the questionnaire mixed model, comprising dichotomous questions, Likert scale questions, multiple-choice questions, and others. The questionnaire items were carefully selected to correspond with the study objectives, guaranteeing a thorough investigation of relevant elements. Clarity was given great priority during the selection process, with the goal of crafting questions that accurately capture the necessary data and promote a sophisticated comprehension of the research issue. This careful approach promotes transparency and coherence in addressing the stated objectives, which strengthens the study’s validity.

A total of 31 items were included for the survey, plus one item dedicated to informed consent. Two items were excluded as it is a sub or additional item to the current context. Seven items are related to demographic characteristics, one item is related to the type of facemask used. The other items included in the questionnaire were classified and examined the various domains related to use and disposal of face masks such as, washing hands before and after using facemasks (two items), correctly wearing facemasks (five items), sharing masks with others (one item), removing masks during social or personal meetings (two items), efficacy and reuse of medical facemasks (three items), proper disposal practice (four items), consequences of incorrect disposal practices (two items) and proper disposal technique (two items). The responses to the domains were categorized into two categories such as positive and negative.

The correct/positive responses to the individual items were scored 1 and wrong/negative responses to the individual items were scored 0. In addition to that, missing responses or median level responses were given with lower scores (score 0). The scores of items included in each domain were summed up. More than or equal to 50% of the total score in each domain were considered positive and correct and the remaining were considered negative. While performing statistical analysis the missed demographic values were replaced by using statistical package for social sciences (SPSS), version 22.0 for Windows.

The content validity was estimated by a pre-test, and the internal consistency was calculated. The calculated Cronbach’s alpha value was 0.68. The questionnaire was uploaded to Google Forms, and the link was distributed electronically and *via* various social media platforms.

#### Ethical considerations

This research was approved by the research ethics committee of King Khalid University (ECM#2020-3204). The data was kept confidential and all the participants participated voluntarily by providing electronic consent.

#### Statistical analysis

The data were imported as an Excel file from Google Forms. The Excel format data were exported to the statistical package for social sciences (SPSS), version 22.0 for Windows for statistical analysis. Descriptive statistics were used to illustrate the demographic characteristics. Binomial logistic regression analysis was used to determine the relationship between demographic characteristics and the responses. Odds ratios were calculated and presented with 95% confidence intervals. The binomial logistic regression can describe the link between a binary result and predictor factors, which is consistent with the categorical response variable of the study, it was selected for the analysis. Because the linearity and independence assumptions were satisfied, the analysis’s robustness was guaranteed. This approach was chosen in order to identify important factors in accomplishing the goals of the study, which included comprehending and forecasting binary outcomes.

## Results

### Demographic characteristics

The number of participants included in the analysis were 198, and their demographic details are provided in Table 1. A total of 73% of the participants included in the current survey were male. Most participants (45%) were 18–25 years of age. A total of 89% of the participants included in the current survey were living with family. A total of 85% of the participants had completed college or university-level education, and no participants were illiterate; 42% were employed, 37% were students, 15% were unemployed, and 6% were homemakers. A total of 45% of the participants earned less than 3,000 Saudi riyals per month, and 43% were earned more than 8,000 Saudi riyals per month. Of the participants and their family members, 43% had a history of COVID-19 infection. A total of 62 & 22% of the current study participants used medical or surgical masks and cloth/fabric masks, respectively. The remaining participants used N95 facemasks.

**Table 1:** Demographic characteristics of the study subjects.

Characteristics		Frequency	Percent	
Age group in years	18–25	89	45	
	26–35	41	21	
	36–45	21	11	
	45–50	20	10	
	More than 50 years	25	13	
Gender	Male	143	73	
	Female	53	27	
Living status	Alone	21	11	
	Family	177	89	
Education	Secondary school	29	15	
	College/University	169	85	
Occupation	Student	72	37	
	Home maker	11	6	
	Working	83	42	
	Not working	30	15	
Monthly income (SAR)	Less than 3,000	83	45	
	3,000–6,000	14	7	
	6,000–8,000	9	5	
	More than 8,000	81	43	
Covid diagnosis history –individual or family	Negative	113	57	
	Positive	84	43	

The results from the binary logistic regression analysis, which examined the factors that tended to affect various domains of usage of facemasks among the current study participants are shown in Table 2.

**Table 2:** Binary logistic regression analysis testing the impact of demographic characteristics on various domains of usage of face masks.

Characteristics		Washing hands before and after using facemasks				Correctly wearing facemasks				Sharing masks with others				Removing masks during social or personal meetings			
		OR 95% C.I.		Sig.		OR 95% C.I.		Sig.		OR 95% C.I.		Sig.		OR 95% C.I.		Sig.
Age in years	18–25	**Reference**				**Reference**				**Reference**				**Reference**			
	26–35	.641 (.123–3.34)		.598		1.620 (.24–0.63)		.615		.000 (0.000)		.998		.000 (0.000)		.998	
	36–45	1.618 (.371–7.05)		.522		.448 (.085–2.35)		.343		.000 (0.000)		.998		.000 (0.000)		.998	
	45–50	1.211 (.243–6.03)		.815		.820 (.126–5.33)		.835		.000 (0.000)		.998		.000 (0.000)		.998	
	>50 years	1.534 (.327–7.20)		.588		1.167 (.15–8.82)		.881		2.121 (0.000)		1.000		.000 (0.000)		.998	
Gender	Male	**Reference**				**Reference**				**Reference**				**Reference**			
	Female	.431 (.191–.972)		.042^a^		.934 (.378–2.30)		.882		.295 (.031–2.77)		.286		.000 (0.000)		.997	
Living status	Alone	**Reference**				**Reference**				**Reference**				**Reference**			
	Family	1.658 (.565–4.87)		.358		1.100 (.32–3.76)		.880		.426 (.081–2.24)		.315		.246 (.041–1.49)		.128	
Educational status	Secondary school	**Reference**				**Reference**				**Reference**				**Reference**			
	College/University	.914 (.332–2.51)		.861		.874 (.287–2.66)		.813		.964 (.079–11.71)		.977		.510 (.075–3.48)		.492	
Occupation	Student	**Reference**				**Reference**				**Reference**				**Reference**			
	Homemaker	5.196 (1.21–22.2)		.026^a^		.416 (.088–1.96)		.268		.000 (0.000)		.998		.097 (.008–1.23)		.072^b^	
	Working	1.059 (.155–7.23)		.954		1.077 (.14–7.86)		.942		.000 (0.000)		.997		000 (0.000)		1.000	
	Not working	.704 (.206–2.409)		.576		1.227 (.29–5.09)		.778		.000 (0.000)		.998		.533 (.01–15.45)		.714	
Monthly income in Saudi Riyals	Less than 3,000	**Reference**				**Reference**				**Reference**				**Reference**			
	3,000–6,000	.604 (.195–1.86)		.382		.400 (.109–1.46)		.165		.402 (.020–8.18)		.553		5.749 (.33–98.5)		.228	
	6,000–8,000	2.919 (.55–15.31)		.205		.776 (.11–5.092)		.792		.621 (.02–15.25)		.770		14.4 (.85–245.7)		.065^b^	
	>8,000	3.56 (.640–19.88)		.147		.339 (.056–2.04)		.238		(0.000)		.999		(0.000)		.999	
COVID diagnosis history	Negative	**Reference**				**Reference**				**Reference**				**Reference**			
	Positive	1.617 (.779–3.35)		.197		1.69 (.768–3.73)		.191		2.140 (.45–9.99)		.333		1.355 (.34–5.32)		.664	

**Notes.**

CI Confidence Interval

* *P* < 0.01.

a *P* < 0.05.

b *P* < 0.1.

### Usage of facemasks

#### Washing hands before and after using facemasks

The data provided cover the characteristics of the individuals who wash their hands before and after using facemasks, including variables, such as age, gender, living status, educational status, occupation, monthly income, and COVID diagnosis history.

In terms of age, the reference group is individuals aged 18–25 years. The aged 26–35 years had lower odds of positive attitudes toward the frequent washing of hands before and after using facemasks (OR =0.641, 95% CI [0.123–3.34]). Similarly, individuals aged 36–45, 45–50, and over 50 years had ORs of 1.618 (95% CI [0.371–7.05]), 1.211 (95% CI [0.243–6.03]), and 1.534 (95% CI [0.327–7.20]), respectively.

Gender played a significant role in the attitude toward washing hands before and after using facemasks (*P* = 0.042). Considering the male gender as a reference, the female gender had significantly lower odds of a positive attitude toward washing hands before and after using facemasks (OR =0.431, 95% CI [0.191–0.972]). In terms of living status, individuals living with their families were more likely than those living alone to wash their hands before and after using facemasks (OR =1.658, 95% CI [0.565–4.87]). Occupation was found to significantly impact the attitude toward washing hands before and after using facemasks, where homemakers had a much higher positive attitude than those in other occupations (OR =5.196, 95% CI [1.21–2.22]). Regarding educational status, the reference group is individuals with a secondary school education. The data show that individuals with a college/university education have lower odds of washing their hands before and after using facemasks (OR = 0.914, 95% CI 0.332 to 2.51). The *p*-value for this comparison is 0.861. A higher monthly income tends to increase the positive attitude toward hand washing before and after using a facemask. COVID-19 diagnosis status among the participants or to anyone to their family was not found to impact the attitude toward hand washing before and after using facemasks.

#### Correctly wearing facemasks

The participants 26–35 years of age were found to wear their facemasks properly (OR =1.620, 95% CI [0.24–0.63]. None of the other demographic characteristics impacted the knowledge about or attitude toward the on proper wearing of facemasks.

#### Sharing facemasks with others

The results of our study indicated that neither age group nor occupational status affected the odds of sharing facemasks. The results also showed that most of the participants in our study did not share their facemask with others, and no demographic characteristics changed their attitude (*P* > 0.05).

#### Removing masks during social or personal meetings

A negative attitude toward removing facemasks during social or personal meetings was observed among the participants staying with family (OR = 0.246, 95% CI [0.041–1.49]), and those who had a college- or university-level education (OR =0.510, 95% CI [0.075–3.48]). The participants with a monthly income range of 6,000–8,000 Saudi riyals (OR =14.4, 95% CI [0.85–245.7]) and a positive history of COVID-19 infection (OR =1.355, 95% CI [0.34–5.32]) had a high positive attitude toward not removing facemasks during their social or personal meeting.

The results of the binary logistic regression analysis, examining the factors affecting knowledge and practice regarding the disposal of used facemasks, are shown in Table 3.

**Table 3:** Impact of demographic characteristics on knowledge and practice of face maks disposal.

Characteristics		Efficacy and reuse of medical facemasks		Proper disposal practice		Consequences of incorrect disposal of facemasks		Proper disposal technique	
		OR 95% C.I.	Sig.	OR 95% C.I.	Sig.	OR 95% C.I.	Sig.	OR 95% C.I.	Sig.
Age in years	18–25	**Reference**		**Reference**		**Reference**		**Reference**	
	26–35	.202 (.032–1.298)	.092^b^	2.30(.481–11.01)	.297	3.12 (.085–15.33)	.536	2.11 (.25–17.47)	.486
	36–45	.861 (.159–4.655)	.862	1.46(.354–6.034)	.600	.623 (.023–17.08)	.780	.279 (.046–1.68)	.163
	45–50	.950 (.160–5.640)	.955	.142 (.014–1.426)	.097	.061 (.002–2.084)	.120	.218 (.031–1.55)	.128
	>50 years	1.586 (.271–9.28)	.609	.200 (.032–1.251)	.085	(0.000)	.998	.179 (.029–1.11)	.066^b^
Gender	Male	**Reference**		**Reference**		**Reference**		**Reference**	
	Female	.067 (.024–.187)	.000^*^	.734 (.282–1.912)		.527	.408 (.075–2.232)	.301	1.25 (.443–3.54)	.670
Living status	Living alone	**Reference**		**Reference**		**Reference**		**Reference**	
	Family	.838 (.210–3.342)	.802	1.22 (.327–4.544)	.767	2.291 (.24–21.85)	.471	.975 (.241–3.93)	.972
Educational status	Secondary school	**Reference**		**Reference**		**Reference**		**Reference**	
	College/University	5.356 (1.74–16.40)	.003*	2.21 (.732–6.719)	.159	.378 (.081–1.755)	.214	1.94 (.392–9.67)	.415
Occupation	Student	**Reference**		**Reference**		**Reference**		**Reference**	
	Homemaker	3.046 (.545–17.03)	.205	.60 (.137–2.634)	.499	.139 (.011–1.800)	.131	.463 (.084–2.54)	.376
	Working	.357 (.040–3.160)	.355	.00 (0.000)	.999	.000 (0.000)	.998	2.46 (.16–36.84)	.513
	Not working	1.765 (.402–7.74)	.451	3.60 (.940–13.79)	.061	6.41 (.296–139.0)	.236	2.61 (.52–13.03)	.240
Monthly income in Saudi riyals	Less than 3,000	**Reference**		**Reference**		**Reference**		**Reference**	
	3,000–6,000	1.913 (.542–6.74)	.313	1.45 (.394–5.348)	.576	.707 (.059–8.429)	.784	.966 (.239–3.91)	.962
	6,000–8,000	8.95 (1.17–68.10)	.034^a^	.252 (.033–1.915)	.183	.188 (.010–3.416)	.259	.000 (0.000)	.998
	>8,000	1.80 (.230–14.10)	.575	3.24 (.435–24.24)	.251	.065 (.002–1.991)	.117	.000 (0.000)	.999
COVID diagnosis history	Negative	**Reference**		**Reference**		**Reference**		**Reference**	
	Positive	.573 (.254–1.293)	2c.180	2c1.50 (.638–3.561)	.350	1.06 (.292–3.862)	.927	.308 (.113–.837)	.021^a^

**Notes.**

CI Confidence Interval

* *P* < 0.01.

a *P* < 0.05.

b *P* < 0.1.

### Disposal of used facemasks

#### Efficacy and reuse of medical facemasks

Approximately 62.12% of the participants in our survey used medical or surgical facemasks. The participants aged 50 years and above had good knowledge concerning the efficacy and reuse of facemasks. Educational status tended to significantly impact the knowledge about and reuse of facemasks (*P* = 0.003), where those who had completed college- or university-level education had a high level of knowledge (OR = 5.356, 95% CI [1.74–16.40]). In addition, there was a significant difference in terms of knowledge about the efficacy and reuse of facemasks between male and female participants (*P* = 0.000). Living status, occupation, income, or positive COVID-19 diagnosis history did not influence knowledge about the efficacy and reuse of facemasks.

#### Proper disposal practice

None of the demographic characteristics influenceed the practice of facemask disposal. The participants aged 26–35 years (OR = 2.30, 95% CI [0.481–11.01]), those living with family (OR = 1.22, 95% CI [0.327–4.544]), those who had a college- or university-level education (OR = 2.21, 95% CI [0.732–6.719]) and those with high monthly income (OR = 3.24, 95% CI [0.435–24.24]) were found to dispose of their used facemasks properly.

#### Consequences of incorrect disposal of facemasks

Similar to proper disposal practice, none of the demographic characteristics influenced the participants’ knowledge about the consequences of using the wrong method of disposal for used facemasks. Most of the categories in the demographic characteristics had low knowledge about the consequences of incorrectly disposing of facemasks (OR<1).

#### Proper disposal technique

The participants aged 18–25 years had better knowledge than others about proper facemask disposal techniques. Females (OR = 1.25, 95% CI [0.443–3.54]), and participants with a college- or university-level education (OR =1.94, 95% CI % 3.92–9.67) had a positive attitude toward proper disposal practices. No demographic characteristics impacted the knowledge about and practice of proper facemask disposal.

## Discussion

Respiratory infections are not new. From seasonal flu to catastrophic outbreaks such as severe acute respiratory syndrome (SARS) in 2006, H1N1 in 2009, SARS-like disease in the Middle East in 2012 and COVID-19 since the end of 2019, respiratory infections have been a threat worldwide. Their reemergence or evolution of new pathogens may cause future pandemics (Sim, Moey & Tan, 2014)**.** It has been reported that zoonotic events caused by the introduction of viruses into humans from mammals likely to lead to the next pandemic (Neumann & Kawaoka, 2023). Facemasks have been regarded as one of the major strategies to prevent the transmission of respiratory pathogens in the past and at present (Babatola et al., 2023) and they will continue to be used in future. During the COVID-19 pandemic, it was made mandatory to use facemasks in the Kingdom of Saudi Arabia. Extensive use of these facemasks necessitates their appropriate use; otherwise, they could become a biohazard (Al Naam et al., 2021). Hand hygiene ought to be observed prior to putting on the facemask and subsequent to its removal since this practice can reduce inadvertent contact between the face and potentially contaminated hands. Such precautions preserve the cleanliness of our environment and safeguard against other infections (Kampf & Kramer, 2004; Wangchuk et al., 2023). As seen in our study, those aged 26 to 35 years showed excellent handhygiene practices when using facemasks, surpassing the other age groups could be owed to the reason that the younger generation have a better access to the informatin, awareness, education through the technical advancement and social and peer awareness and goes similar in findings with the study published among university students from Abudhabi (Ajaj et al., 2023).

It has been inferred that older adults do not consistently adhere to good hand hygiene practices, which is directly linked to a lack of health literacy (Or, Wong & Chung, 2020). This finding suggest that older individuals may require additional education to ensure they fully understand and practice hand hygiene protocols. Males showed a more positive attitude than females in our study, in contrast with several previous studies that have revealed different obvious gender distinction regarding the perception and effectiveness of hand hygiene (Rubin et al., 2009; Park et al., 2010). Associated with this difference in knowledge levels, it has been postulated that females are less likely to take risks and thus more likely to follow hand-washing recommendations (Sim, Moey & Tan, 2014).

Participants with low monthly income and a history of COVID-19 infection showed a negative attitude toward frequent hand washing before and after using using facemasks. These participants were possibly unaware that hand hygiene is an important health measure before and after facemask use. A similar situation was observed during the SARS outbreak in 2002–2003, whe some hand hygiene behaviors were not respected, even by hospital workers (Lau et al., 2004).

Facemasks use has been recommended worldwide to prevent the spread of COVID-19 infection (Fouladi Dehaghi et al., 2020; Tabatabaeizadeh, 2021). The appropriate use of facemasks is crucial for containing the transmission of respiratory droplets and protecting oneself and others from infectious diseases. It is important to follow recommended guidelines for wearing facemasks correctly and consistently. In this study, the participants aged 26–35 years were found to wear their facemasks properly. However the study finding also suggests that demographic characteristics can negatively influence the appropriate use of facemasks. This might be due to the relatively simple designs of facemasks, leading many people to assume that they know how to use them (Ho, 2012)). This assumption can reduce the public’s desire to learn correct facesmask use protocols. In addition, a lack of knowledge might lead to inappropriate facemask use. The findings of our study are in line with those of a previous study that revealed an association between poor COVID-19 knowledge and a deficiency in facemask use (Al-Hanawi et al., 2020). We also found a significant association between facemask use attitudes and level of education, which is in agreement with similar studies conducted in Saudi Arabia and Bangladesh, that demonstrated that high educational qualifications resulted in positive attitudes toward COVID-19 preventive measures (Ferdous et al., 2020; Al-Hanawi et al., 2020).

Sharing masks can increase the risk of spreading infections between individuals since respiratory droplets containing pathogens can accumulate on the mask’s surface. Therefore, individuals should not share their masks with others and should be cautious about removing facemasks during social and personal meetings (World Health Organization, 2020). In the current study, most of the participants did not share their facemask with others. Participants staying with family, those with a college-or university-level education, and those who were working demostrated negative attitudes toward removing masks during their social or personal meetings. Therefore, information regarding the dangers of sharing masks or removing them during their social or personal meetings and how avoiding these actions helps prevent infection spread must be strognly conveyed to everyone.

It is interesting to note that, participants with a monthly income of SR 6,000–8,000 and a positive history of COVID-19 infection had a highly positive attitude toward not removing facemasks during their social or personal meetings. This finding suggests and effct of their previous experience with the disease or awareness of the dangers of removing masks. The effectiveness of infection control methods may be negatively impacted by sharing or taking off face masks during social or private interactions. People need to be aware of the possible dangers and repercussions that come with these actions. Therefore, it is necessary to post a warning reminding people not to take off their face masks in public areas. Additionally, educational initiatives should be put in place to inform people about the value of adhering to infection control protocols and good hygiene practices. These educational initiatives shallbe augmented by structured information leaflets or printed education materials, as these education materials are frequenlty used resource for education (Easwaran et al., 2023; Vigneshwaran, Padmanabha & Devanna, 2013).

During the COVID-19 pandemic, mask decontamination and reuse have been considered last-resort strategies during periods of mask shortages (Bhattacharjee et al., 2020). Participants belonging to the aged 50 years and older and with higher education had high knowledge regarding disinfection and reuse of facemasks. The most common methods used to disinfect and sterilize masks are decontamination with hot water or steam. Notably, this method may not be effective for all types of masks (Fathizadeh et al., 2020; Ma et al., 2020).

Improper facemask disposal is increasing in frequency, which contirbutes to infections. When worn or discarded incorrectly, facemasks can become contaminated and serve as a potential source of infection (Shetty et al., 2020; Mudenda et al., 2020; Mudenda et al., 2021). The adult population and participants with a high level of education were found to dispose of their used facemasks properly following the appropriate recommended procedures. The proper disposal of used facemasks is important for long-term care facilities or households with elderly and immune-compromised individuals, where improperly discarded masks can lead to severe repercussions (Bhattacharjee et al., 2020; Kaewchutima et al., 2023). The intitiatives from the educational institutions is imperative that they can take the lead in offering instruction on the subject. The incorrect disposal of facemasks poses a risk to the environment and could accelerate the spread of infection among people particulary among young people. According to the current study results, those aged 50 years and older should be targeted for education regarding the proper disposal of used facemasks and trained on how to collect, pack, and dispose of used facemasks correctly.

Our findings highlighted an important problem regarding how to use and/or reuse the facemasks or dispose of the used facemasks correctly. Disinformation, incorrect opinions, and knowledge related to mask use and reuse may increase the infection risk in the community. As reported, incorrectly disposed facemasks could become biohazards and risk new infections and outbreaks (Al Naam et al., 2021). Hence, awareness and information campaigns aimed at the general population are needed to implement the correct use of masks and limit the infection rate as much as possible. This could help in prevent new outbreaks and avoid the reemergence of COVID-19 outbreaks.

## Limitations

Recognizing the limitations of facemask usage and disposal evaluation will guide future research, potentially leading to more extensive investigations on these essential infection control issues. The present research used non-probabilistic conveniece sampling, which limits its applicability to the entire population and may under- or overrepresent it. The sampling technique, small sample size and regional differences in Saudi Arabia may potentially limit the generalizability of the findings. This could have an impact on the results’ capacity to be applied to larger populations. Alternative sample techniques should be used in future studies to gain a more thorough insight. The small sample size was due to difficulties recruiting a large number of participants due to time, resources, or the study community. Depending on the research design and analytic methodologies, a small sample size can yield useful insights and statistical significance. Self-reported, voluntary electronic survey data were obtained. Thus, agreement bias may have altered the responses. The current study design limits causal inference. The current study identified determinants of facemask use and disposal but did not evaluate proper use or disposal. Future researchers should adopt stratified or cluster sampling to obtain a sample more representative of the population.

Future researchers can also use Bayesian theory, which provides a mathematical technique for revising probability based on new evidence for future research. This approach relies on conditional probability to incorporate past knowledge or beliefs into the analysis.

Machine learning algorithms can be used alongside Bayesian theory to tackle these challenges. These algorithms can examine enormous datasets and find patterns to correct ratios and fields by considering many aspects and variables.

## Conclusions

The present investigation examined a range of demographic factors that impact the accurate use and proper disposal of facemasks. The three main characteristics that are crucial in developing targeted intervention strategies to enhance the prudent use and proper disposal of facemasks are gender, income, and employment. Furthermore, the findings of the present research suggest that it would be beneficial for facemask producers to reconsider the design of their packaging and provide comprehensive instructions pertaining to the appropriate use, and disposal of facemask and accurate application techniques. Critically, alteration of mindset toward recognizing and effectively addressing potential risks requires improvement. Therefore, in order to increase understanding, it is imperative to develop national awareness campaigns and similar initiatives. Health authorities should set up a workable system that allows the general population to gather and discard worn facemasks, beginning with informational campaigns and continuing through the ultimate disposal phase. Moreover, the results of the present study have significant implications for health preventive programs aimed at preparing for future pandemics since they highlight the males, high income and highly educated groups have shown better compliance and females, low income and not high educated groups should be prioritized in the development of such policies. Furthermore, it is advisable to integrate these interventional initiatives into national health policies to improve preparedness for future pandemics.

##  Supplemental Information

10.7717/peerj.16889/supp-1 Supplemental Information 1 Raw data of the participants with consent

10.7717/peerj.16889/supp-2 Supplemental Information 2 Questionnaire

## Additional Information and Declarations

### Data Availability

The following information was supplied regarding data availability: The raw measurements are available in the Supplemental file 1.
