# Supplementary material for: Examining factors influencing public knowledge and practice of proper face mask usage during the COVID-19 pandemic: a cross-sectional study
Source: PeerJ. 2024 Feb 8;12:e16889. doi: 10.7717/peerj.16889 (PMC10859079; doi:10.7717/peerj.16889)
Supplement: Supplemental Information 2 [file peerj-12-16889-s002.pdf]

Exploring determinants towards usage and disposal of face mask during the covid pandemic among common public

Dear Participant,
Greetings of the day,

During the current pandemic situation, usage of masks are important for safety of ourselves and others.
This survey intend to identify the determinants and to evaluate the knowledge and attitude on the usage and disposal of face masks. This survey will take only 3-5 minutes to complete. And deals with basic questions related to the usage and disposal of face masks.
PARTICIPATION - Your participation in this survey is 100% voluntary.
BENEFITS - You will receive no direct benefits from participating in this research study. However, your responses may help us in scientific research and community benefits.
RISKS - There are no foreseeable risks involved in participating in this study other than those encountered technically in day-to-day life.
CONFIDENTIALITY - Your survey answers will be sent to us and the data will be stored in a protected electronic format. We do not collect identifying information such as Name, IP addresses or locations and etc.
Therefore, your responses will remain Safe. No one will be able to identify you or your answers, and no one will know whether or not you participated in the study other than the Researcher.
عزيزي المشترك,
تحية طيبة وبعد,
نرجو أن تكونوا بأمان وصحة وعافية.
ان استخدام الاقنعة الواقية للوجه (الكمامات) خلال فترة كورونا بالغة الاهمية للتقليل بإذن الله من انتقال الفيروس بين الناس. ولهذا, سيأخذ هذا الاستبيان من وقتك من 3 - 5 دقائق للاجابة على أسئلته المتعلقة باستخدام الاقنعة والتخلص منها. إن موافقتك للمشاركة في هذا الاستبيان هي طواعية وتكرما منك بشكل كامل. وإن التعامل مع بياناتك سيتم بشكل يضمن سريتها خلال فترات البحث كما نحيطك علما أن الباحثين لن يقوموا بسؤالك عن هويتك او اسمك او عنوانك خلال هذه الاسئلة. ان مشاركتك في هذا الاستبيان قيمة ومفيدة.

* Indicates required question

1. ELECTRONIC CONSENT: Clicking on the “Agree” button indicates that, you have read the above information, you voluntarily agree to participate, you are 18 years of age or older• If you disagree NO NEED to fill the survey. الموافقة الالكترونية للمشاركة: عند موافقتك للمشاركة في البحث الرجاء التكرم بالاختيار ب"موافق" للموافقة والاستمرار في عرض الاسئلة, أو "غير موافق" عند عدم الموافقة على المشاركة *

Mark only one oval.

- Agree موافق
- Disagree غير موافق

General information معلومات عامة

2. Age in years العمر بالسنوات

Mark only one oval.

- 18 - 25
- 26 - 35
- 36 - 45
- 45 - 50
- More than 50 أكثر من 50 سنة

3. Gender الجنس

Mark only one oval.

- Male ذكر
- Female انثى

4. Living status الحالة الاجتماعية

Mark only one oval.

- Alone وحيدا
- With family مع عائلة

5. Education التعليم

Mark only one oval.

- No formal education غير متعلم
- Primary school ابتدائي
- Secondary school ثانوي
- College/University جامعي أو أعلى

6. Occupation الوظيفة

Mark only one oval.

- Student طالب
- Home maker ربة منزل
- Working يعمل
- Not working لا يعمل

7. Monthly Income (Saudi Riyal) الدخل الشهري

Mark only one oval.

- Less than 3000 أقل من 3000 ريال
- 3000-6000
- 6000-8000
- More than 8000 أكثر من

8. Current status of your occupation الوضع الحالي للعمل بسبب الجائحة

Mark only one oval.

- Work from home العمل من المنزل
- Work at office/field العمل من المكتب/الميدان

9. You or any of your family member tested positive for COVID 19: هل سبق وأن تم اصابتك او احد من العائلة كورونا من قبل؟

Mark only one oval.

- Yes نعم
- No لا

10. Are you working as health care professional? هل تعمل كممارس صحي؟

Mark only one oval.

- Yes نعم
- No لا

Untitled Section

11. What kind of face mask do you use? أي من الانواع التالية لاقنعة الوجه تقوم باستخدامها؟

Mark only one oval.

- Cloth or Fabric mask القناع القماشي
- Medical/Surgical face mask القناع الطبي/الجراحي
- N 95 mask قناع N 95 الطبي

Usage of face mask استخدام اقنعة الوجه

12. 1. Do you wash your hands before start using face mask? هل تقوم بغسل يدك قبل استخدام القناع؟

Mark only one oval.

- Yes نعم
- No لا
- Sometimes أحيانا

13. 2. While using face mask, do you uncover any one of the following? Face or chin or nose. عند استخدامك للقناع, هل تقوم بعدم تغطية الوجه او الفك او الانف؟

Mark only one oval.

- Yes نعم
- No لا

14. 3. Do you frequently touch the face mask, preferably on the front side? هل تقوم بلمس جانب وجهك الامامي باستمرار خلال لبس الكمامة؟

Mark only one oval.

- Yes نعم
- No لا

15. 4. If you are using cloth/fabric mask, do you wash the mask with the detergent when you want to reuse it? اذا كنت تستخدم الكمامة/القناع القماشي, هل تقوم بغسله بالمنظفات او المطهرات قبل اعادة استخدامه؟

Mark only one oval.

- Yes نعم
- No لا

16. If yes to question number 4, how often do you wash your Cloth/Fabric mask?اذا كانت اجابتك للسؤال الرابع ب"نعم", كم مرة تقوم بغسل الكمامة القماشية قبل استخدامها؟

Mark only one oval.

- Everyday كل يوم
- Every alternative day يوم بعد يوم
- Weekly once مرة في الاسبوع
- If exposed to a crowded area اذا تعرضت لزحام

17. 5. Do you wash your hands after removing the face mask? هل تقوم بغسل يديك قبل خلع الكمامة القماشية؟

Mark only one oval.

- Yes نعم
- No لا

18. 6. Do you wear a loose mask? هل تقوم بلبس كمامة غير مشدودة/ مرتخية

Mark only one oval.

- Yes نعم
- No لا

19. 7. Do you share your mask with others? هل تقوم بمشاركة كمامتك مع الاخرين؟

Mark only one oval.

- Yes نعم
- No لا

20. 8. Do you wear a mask under the nose? هل تقوم بوضع كمامتك تحت أنفك؟ ويبقى الانف مكشوفا؟

Mark only one oval.

- Yes نعم
- No لا

21. 9. Do you remove mask when you are talking with unknown person?هل تقوم بخلع الكمامة عند التحدث مع شخص لا تعرفه؟

Mark only one oval.

- Yes نعم
- No لا

22. 10. Do you remove mask when you are talking with your friends or office colleagues?هل تقوم بخلع الكمامة عند التحدث مع الاقارب او الاصدقاء او الزملاء؟

Mark only one oval.

- Yes نعم
- No لا

23. 11. Do you reuse the Medical/Surgical mask? هل تقوم باستخدام الكمامة الجراحية/الطبية أكثر من مرة؟

Mark only one oval.

- Yes نعم
- No لا

24. 12. If you use Medical/Surgical mask, do you ensure colored side faces outwards? عند استخدام الكمامة الطبية, هل تتأكد من وجود الجانب الملون للخارج؟

Mark only one oval.

- Yes نعم
- No لا

Disposal of face masks التخلص من الكمامات

25. 13. Approximately, How long the Medical/Surgical face masks can be used? تقريبا, كم مدة استخدامك للكمامة الطبية او الجراحية؟

Mark only one oval.

- Less than 6 hours أقل من 6 ساعات
- 6 - 8 hours من 6 الى 8 ساعات
- 8 - 12 hours من 8 الى 12 ساعة
- 24 hours ل24 ساعة

26. 14. While you remove your mask, will you touch front side of the mask with your bare hands? عند خلع الكمامة, هل تقوم بملامسة الجانب الامامي من الكمامة بيديك؟

Mark only one oval.

- Yes نعم
- No لا

27. 15. Do you use gloves or tissues to remove face mask? هل تستخدم القفاز عند خلع الكمامة؟

Mark only one oval.

- Yes نعم
- No لا

28. 16. Do you throw your face mask in an open environment or in open trash bin after its usage? هل تقوم برمي الكمامة بعد استخدامها في مكان عام او في سلة القمامه العادية ؟

Mark only one oval.

- Yes نعم
- No لا

29. 17. Do you discard the face mask, if it is wet or damaged? هل تقوم برمي الكمامة اذا وجدتها مبللة او ممزقة؟

Mark only one oval.

- Yes نعم
- No لا

30. 18. Do you think improperly discarded face masks has potential health hazards? هل تعتقد ان رمي الكمامات الغير صحيح وفي غير اماكنها المخصصة خطر على الصحة وقد ينقل الفيروس؟

Mark only one oval.

- Yes نعم
- No لا

31. 19. Do you think pathogens can survive for long hours in used face masks? هل تعتقد ان الميكروبات تعيش على الكمامات المستخدمة لفترة طويلة؟

Mark only one oval.

- Yes
- No لا

32. 20. After usage, do you leave your mask within the reach of others? بعد الانتهاء من استخدام الكمامة, هل تقوم بوضعها في مكان يسهل الوصول اليه؟

Mark only one oval.

- Yes نعم
- No لا

33. 21. Do you fold inwards (possibly several folds) and discard your mask to avoid the pathogens being exposed out side? هل تقوم بثني الكمامة مرات عديدة قبل رميها لتجنب انتقال الميكروبات للخارج؟

Mark only one oval.

- Yes نعم
- No لا

This content is neither created nor endorsed by Google.

Google Forms
